# The Bioavailability of Soluble Cigarette Smoke Extract Is Reduced through Interactions with Cells and Affects the Cellular Response to CSE Exposure

**DOI:** 10.1371/journal.pone.0163182

**Published:** 2016-09-20

**Authors:** Jeffrey S. Bourgeois, Jeeva Jacob, Aram Garewal, Renata Ndahayo, Julia Paxson

**Affiliations:** Department of Biology, College of the Holy Cross, Worcester, MA, 01610, United States of America; Augusta University, UNITED STATES

## Abstract

Cellular exposure to cigarette smoke leads to an array of complex responses including apoptosis, cellular senescence, telomere dysfunction, cellular aging, and neoplastic transformation. To study the cellular response to cigarette smoke, a common in vitro model exposes cultured cells to a nominal concentration (i.e. initial concentration) of soluble cigarette smoke extract (CSE). However, we report that use of the nominal concentration of CSE as the only measure of cellular exposure is inadequate. Instead, we demonstrate that cellular response to CSE exposure is dependent not only on the nominal concentration of CSE, but also on specific experimental variables, including the total cell number, and the volume of CSE solution used. As found in other similar xenobiotic assays, our work suggests that the effective dose of CSE is more accurately related to the amount of bioavailable chemicals per cell. In particular, interactions of CSE components both with cells and other physical factors limit CSE bioavailability, as demonstrated by a quantifiably reduced cellular response to CSE that is first modified by such interactions. This has broad implications for the nature of cellular response to CSE exposure, and for the design of in vitro assays using CSE.

## Introduction

Cell and tissue damage associated with cigarette smoke exposure continues to be a leading cause of morbidity and mortality globally [1,2]. Exposure to cigarette smoke has been associated with an increased risk of cancer, coronary and vascular diseases, complications during pregnancy, increased lower respiratory tract infections, and chronic lung diseases [3]. The pathophysiology of these pulmonary diseases is multifactorial, and many different cell types are affected [4,5]. Therefore, understanding the cellular response after exposure to cigarette smoke is important, and is studied using both in vivo and in vitro models [6,7]. Cellular responses to cigarette smoke are complex, and are reported to include MAPKs/STAT1-mediated apoptosis, cellular senescence secondary to induced telomere dysfunction and cellular aging, and epigenetic changes associated with neoplastic transformation [8–10].

Cigarette smoke is generated by the combustion, pyrolysis and associated chemical reactions resulting from burning tobacco, and exposes the smoker to upwards of 4000 different xenobiotic chemicals [11,12]. Cigarette smoke contains both gaseous and particulate components, with nicotine, polycyclic aromatics and nitrosamines specifically concentrated in the particulate matter [13]. Smoking a single cigarette deposits between 15–40,000 μg of particulate matter into the respiratory tract [13], and this deposition has been specifically associated with dysregulation of MAPK signaling and MMP1-mediated inflammatory pathways in the lung [14].

In vitro studies to examine cellular response to xenobiotics have become popular, both for the ability of such assays to be easily controlled and manipulated, as well as recent efforts to reduce the use of animals in research [15]. A common in vitro model to study cellular response to cigarette smoke exposure utilizes soluble cigarette smoke extract (CSE). This extract is diluted in growth media and administered as a nominal concentration (i.e. initial concentration) to cultured cells [16–18]. CSE contains both water-soluble chemicals and micro-particulate components of cigarette smoke that are retained after drawing smoke through aqueous solution [16–18]. Recent studies examining the cellular response to CSE exposure suggests that lung cells demonstrate a dose-dependent response to CSE including reduced proliferation, reduced cell viability, and increased apoptosis [8,16]. However, studies using CSE exposure assays differ widely in the concentration and volume of CSE used, and the total number of cells exposed, leading to differences in reported cellular responses [16–18]. There are no reports that examine factors affecting bioavailability of CSE when administered to cultured cells in vitro, and therefore no current explanations for the varied cellular responses seen in CSE exposure assays. However, toxicological studies of other xenobiotics suggest that bioavailability of cytotoxic chemicals can be affected by many variables, including cell binding, cellular metabolism, binding to media components including serum factors, binding to cell culture plastics, xenobiotic degradation and evaporation [19,20].

Our goal in this study was to investigate how specific experimental variables affect cellular response to CSE exposure. We used a variety of functional assays to examine this cellular response to CSE exposure, focusing specifically on cell viability using a standard MTT assay, as well as biomarkers of cytotoxicity using a lactate dehydrogenase release assay and expression of mRNA transcripts associated with cellular cytotoxicity, xenobiotic metabolism, and inflammation. Unexpectedly, we observed that cellular response to CSE exposure is dependent not only on the nominal concentration of CSE (i.e. the initial concentration), but also on the total number of cells present, and on the total volume of CSE solution used. From these observations, we hypothesized that the bioavailability of soluble CSE components is altered by changes in these experimental variables. To test this hypothesis, we used a novel transfer assay in which CSE was exposed to wells containing varying numbers of cells for 60 minutes, and then the resulting potentially depleted CSE was transferred onto a new plate of undamaged cells. This assay confirmed that CSE bioavailability is limited by dose-dependent interactions with cells, as well as by contributions from physical factors that may include variables such as plastic adherence, evaporation and/or degradation. These results are consistent with recent reports in other in vitro xenobiotic assays [19,20]. Furthermore, our analysis of cytotoxicity biomarkers suggests that limitations on CSE bioavailability are accurately reflected in the cellular cytotoxic response. The data we provide in this study have broad implications for the study of cellular responses to cigarette smoke, and raise important questions about CSE bioavailability in in vitro assays.

In addition, we believe that the results of our study are relevant for ensuring accurate and consistent reporting for in vitro CSE studies. As reported for other xenobiotic assays [19], we suggest that developing a standardized reporting protocol is a more accurate dosing metric for in vitro assays examining the cellular response to CSE exposure. In particular, we recommend that standard reporting for in vitro assays examining cellular response to CSE should include nominal concentration of CSE used, number of cells used, total volume of CSE/media used, and the specific cellular responses observed. We believe that this information reported here provides a novel prospective on the cellular response to CSE-induced damage, and is also critical in the future design of in vitro CSE-induced cell damage studies.

## Materials and Methods

### Preparation of soluble cigarette smoke extract

**Fig 1:**
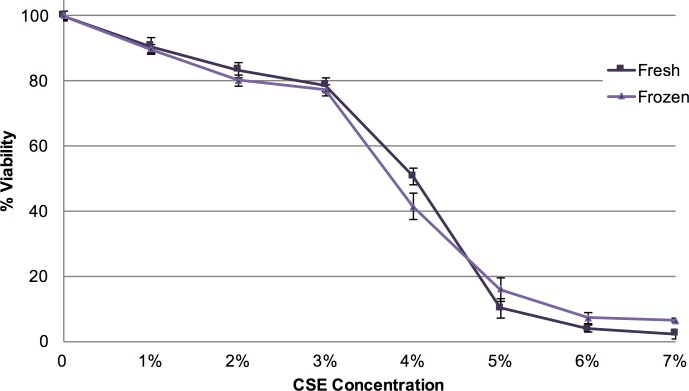
Comparison of cellular response to fresh CSE versus flash-frozen CSE. NIH 3T3 mouse fibroblasts were plated at 15,000 cells/well in a 48 well plate, allowed to grow for 24 hours, exposed to CSE for 24 hours using 400 uL of 0–7% CSE that was either used fresh (within 30 minutes of making) or flash-frozen, then subjected to an MTT viability assay to assess relative cellular damage. Values are reported as % viability compared to cells not exposed to CSE, with error bars representing the standard error of the mean.

3R4F reference cigarettes (Institute of Agriculture, University of Kentucky, USA) were used to prepare soluble cigarette smoke extract (CSE) for this study. Briefly, rubber tubing connected each cigarette to glass tubing submerged in 20 mL of media at the bottom of a 50 mL vacuum filtration flask. Once the cigarette was lit, a vacuum removed air from the flask, drawing cigarette smoke through the media (DMEM, without FBS or antibiotics/antimycotics), and depositing soluble components of the smoke into solution. The vacuum flow was restricted to a constant rate in order to allow the cigarettes to burn at a constant rate of 2 minutes per cigarette. For each 20 mL batch of cigarette smoke extract, two cigarettes were burned for a concentration of one cigarette per 10 mL of media. The CSE made was thoroughly mixed to provide a homogeneous solution and was then filtered through a 0.2 μM sterile filter. Samples were aliquoted, flash frozen in liquid nitrogen, and stored at -80°C until use. Our assays demonstrate that flash freezing does not significantly alter the ability of CSE to reduce cell viability as measured using the MTT assay (**Fig 1**).

### Cell culture

Both NIH 3T3 mouse fibroblasts and NMuMG mouse epithelial cells were cultured in DMEM growth media with high glucose, 10% fetal bovine serum, 1x Gibco Antibiotic-Antimycotic (Thermo Fisher Scientific Inc), and 2mM L-glutamine. Cells were incubated at 37°C, 5% CO_2_ until they reached 70–90% confluence. Cells were lifted using 0.25% TrypLE (Thermofisher cat# 12-604-013), and counted using an inverted light microscope, a hemocytometer, and a 0.4% solution of Trypan Blue in order to assess cell viability.

### Cell viability assay

Cells were plated at the specified number (10,000, 15,000 or 20,000 cells/well) on 48 well cell culture plates, and grown for 24 hours for all experiments unless otherwise noted. Cells were exposed to 400 μL of diluted CSE (unless otherwise stated) and were incubated for 24 hours. All cells were grown in media containing 10% FBS, and the same media was used for the CSE exposure assays (both controls and CSE exposure groups), which may influence the proliferation and/or survival of cells in these assays. Cell viability was then assessed through the yellow tetrazolium MTT (3-(4, 5-dimethylthiazolyl-2)-2,5-diphenyltetrazolium bromide) cell viability and proliferation assay (MTT cell proliferation kit, ATTC catalog # ATCC 30-1010K). Briefly, the cells were incubated for four hours with the yellow MTT reagent, allowing the cells to reduce the reagent, and detecting the amount of the purple formazan byproduct present in the solution after incubation using a spectrophotometric plate reader. The MTT reagent was diluted in complete DMEM media to a concentration of 15 μL MTT reagent per 165 μL, and 165 μL of the diluted solution was added to each well of the 48 well microplate. After four hours, 150 μL of detergent reagent was added to each well, and the plate was allowed to incubate overnight in the dark at 37°C, 5% CO_2_. The absorbance (570 nm) of each well was then measured and recorded. Percent cell viability was calculated by dividing each data point by the average of the unexposed replicates, and multiplying by 100.

### CSE transfer assay

NIH 3T3 fibroblasts were grown at either 25,000 or 50,000 cells/well on 48 well cell culture plates for 24 hours. Different volumes of CSE (either 0.5 mL or 1 mL as specified) were incubated with the cells for 60 minutes. 400 μL of the CSE solution was then carefully removed and transferred to a new 48 well plate with 15,000 cells in each well. These cells were incubated with the 400 μL of transferred CSE for 24 hours before being analyzed using the MTT cell viability assay as described above. Relative bioavailability was calculated using the following equation:

$$Relative\;Bioavalibility=100\%\;\left(\frac{100\%-Vx}{Vb}\right)$$

Where V_x_ is the percent cell viability of each data point, and V_b_ is the average percent viability of the high volume, 0 cell count data point.

### Lactate dehydrogenase (LDH) release assay

Cells were plated at the specified number (10,000, 15,000 or 20,000 cells/well) in 48 well cell culture plates, and grown for 24 hours. Cells were exposed to 400 μL of diluted CSE (0–7%) and were incubated for 24 hours. LDH release was measured using the Pierce LDH cytotoxicity kit (Thermofisher catalog # 88953). Briefly, 50 uL of media was removed from each well, and combined with 50 uL of reaction mixture. This was incubated at room temperature for 30 mins, then 50 uL of stop solution was added. The absorbance was read at 490/680 and LDH release was calculated according to the formula below. Maximum LDH release was obtained by exposing cells to 20% CSE, and spontaneous LDH release was measured in cells exposed to DMEM+10% FBS without CSE:

$$LDH\;release=100\%\;\left(\frac{CSE-exposed\;LDH\;release-spontaneous\;LDH\;release}{Maximum\;LDH\;release-spontaneous\;LDH\;release}\right)$$

### Quantitative PCR

NIH 3T3 fibroblasts were plated on 60 mm plates at either 0.5 x 10^6^ or 1 x 10^6^ cells/plate and grown for 24 hours before exposure to either 3 mL or 6 mL of dilute CSE at one of two nominal concentrations (6% or 8%). After a 24 hour CSE exposure, cells were removed from the plate using TrypLE (Thermofisher cat# 12-604-013), pelleted by centrifugation at 1500rpm for 5 minutes, and then resuspended in 200 μl of RNAprotect Cell reagent (Qiagen cat# 76526). RNA was isolated using an RNeasy plus mini kit (Qiagen cat# 74134), and was quantified using a Nanodrop spectrophotometer. Total RNA was then subjected to genomic DNA elimination and first strand cDNA synthesis using a Superscript IV Reverse Transcriptase kit (Thermofisher cat#18091200). Quantitative PCR was performed on an Applied Biosystems StepOnePlus™ Real-Time PCR thermocycler, using the QuantiNova SYBR Green PCR Kit (Qiagen, cat# 208052) according to the manufacturer’s recommended protocol. Gene specific primers used for the quantitative PCR are listed in **Table 1**. Each sample was analyzed in triplicate, and the relative gene expression was calculated using the comparative ΔΔCT method (Pfaffl, 2001), after normalization to the housekeeping gene Gapdh, which did not show differences in expression between any of the samples analyzed.

**Table 1:** Primers used for quantitative PCR.

Mouse	Primer pairs	Accessoion number	Notes
Cyp1a1	F1 ACGTGAGCAAGGAGGCTAAC	BC125440	Cytochrome 450—toxin metabolism, CSE-induced Lu 2007
	R1 GGCCAAAGCATATGGCACAG		
Gsta2	F1 GAGCTTGATGCCAGCCTTCT	BC061133	Glutathione S-transferase, alpha 2 (Yc2)—toxin metabolism, CSE-induced Lu 2007
	R1 GCATCCAAGGGAGGCTTTCT		
Hmox1	F1 CCTCACAGATGGCGTCACTT	BC010757	Heme oxygenase 1 (dycycling), CSE-induced Lu 2007
	R1 TGGGGGCCAGTATTGCATTT		
Ki67	F1 AGAGGACCCCAAGGAAGTGT	BC053453	Cell proliferation; Mus musculus antigen identified by monoclonal antibody Ki 67
	R1 TGCTTCCTGCTTTGGTGCTA		
Tgfb	F1 AGGGCTACCATGCCAACTTC	BC013738	Myofibroblasts, profibrotic; Mus musculus transforming growth factor, beta 1, mRNA
	R1 CCACGTAGTAGACGATGGGC		
Ptgs2/Cox2	F1 CCAGCACTTCACCCATCAGT	NM_011198	Reported inflammatory mediator up-regulated in fibroblasts after expsosure to cigarette smoke.
	R1 GGGGATACACCTCTCCACCA		
Ccl2/MCP-1	F1 CTGCTGTTCACAGTTGCCG	NM_011333	Reported inflammatory mediator up-regulated in fibroblasts after expsosure to cigarette smoke.
	R1 GCACAGACCTCTCTCTTGAGC		
Gapdh	F1 ACTGAGCAAGAGAGGCCCTA	BC082592	Housekeeping; Mus musculus glyceraldehyde-3-phosphate dehydrogenase, mRNA
	R1 TATGGGGGTCTGGGATGGAA		

### Statistics

Results are expressed as the mean ± SEM. Statistical analyses were performed using analysis of variance (ANOVA) or the unpaired student’ s t test using the Analysis ToolPak add-in for Microsoft Office Excel; p < 0.05 was considered statistically significant. Assumptions for parametric tests were assessed using the IBM SPSS software.

## Results

### Cell viability is affected by changes in nominal concentration of CSE and cell count

We used a standard MTT cell viability assay to assess the cellular response to CSE exposure. To optimize the MTT assay for our experimental design, we first determined the linear range and optimal cell number for use with the MTT reagent for each cell line used, including the NIH 3T3 fibroblasts and NMuMG epithelial cells. This is particularly important for the MTT assay, since cellular metabolism, and therefore MTT metabolism, can be negatively affected by cell-cell contact inhibition and interactions at higher cell counts. We determined that in our 48 well assay, using up to 20,000 cells/well resulted in a linear production of MTT in both cell lines.

**Fig 2:**
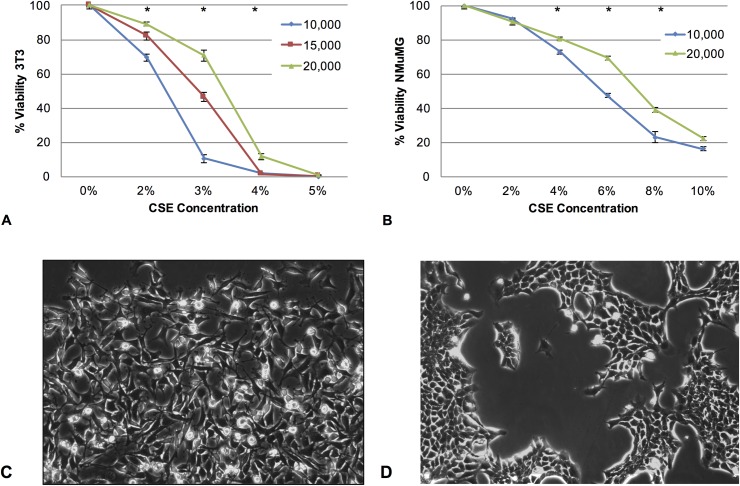
Effects of varying cell type and cell count on cellular response to CSE exposure. **(A)** NIH 3T3 mouse fibroblasts were plated at 10,000, 15,000, or 20,000 cells/well in a 48 well plate, allowed to grow for 24 hours, and exposed to CSE for 24 hours using 400 uL of 0%, 2%, 3%, 4%, or 5% CSE. An MTT viability assay was then used to assess relative cellular damage. (**B**) NMuMG mouse epithelial cells were plated at 10,000 or 20,000 cells/well in a 48 well plate, allowed to grow for 24 hours, and exposed to CSE for 24 hours using 400 uL of 0%, 2%, 4%, 6%, 8% and 10% CSE. An MTT viability assay was then used to assess relative cellular damage. Significant differences in cellular morphology between (**C**) NIH 3T3 mouse fibroblast cells and (**D**) NMuMG mouse epithelial cells are evident in photomicrographs, but do not alter the general trends observed above. In each graph, values are reported as % viability compared to cells not exposed to CSE, with error bars representing the standard error of the mean. * Compared to other cell counts exposed to the same percent CSE, p < 0.05.

We next investigated how cellular response is affected by changes in CSE nominal concentration and cell count. We examined the effect of cell count on the cellular response to CSE exposure by exposing either 10,000, 15,000, and 20,000 3T3 cells (**Fig 2A**) or 10,000 and 20,000 NMuMG cells (**Fig 2B**) to varying concentrations of CSE (0–10%), and compared the viability of the cells after damage to an undamaged control using the MTT assay. Unexpectedly, higher cell numbers for both cell lines showed less of a reduction in percent cell viability compared to lower cell numbers at the same nominal CSE concentration (**Fig 2A and 2B**). For example, at 3% CSE, samples containing 20,000 3T3 cells/well had 71% viability, samples containing 15,000 cells/well had 47% viability, and samples containing 10,000 cells/well had approximately 10% viability. Despite very different cellular morphologies and cell population behaviors between the mesenchymal 3T3 cells and epithelial NMuMG cells (**Fig 2C and 2D**), similar trends were seen after exposure of both 3T3 and NMuMG cells to varying concentrations of CSE, suggesting that this phenomenon is not specific to 3T3 cells, and can be seen equally in both mesenchymal and epithelial cell types. These data are consistent with previous reports demonstrating that the cellular response in other in vitro xenobiotic assays also varies with cell count [19].

### Cellular response is dependent on the volume of CSE solution used

**Fig 3:**
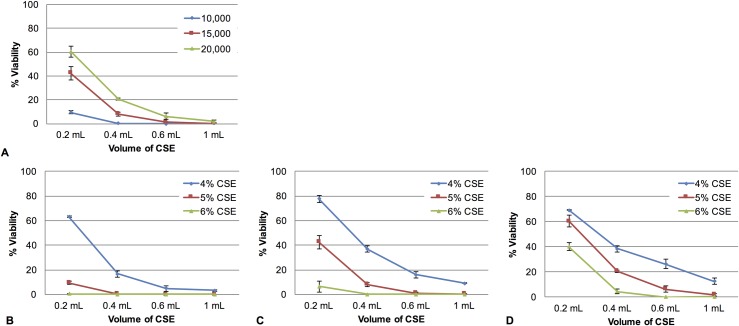
Effects of varying CSE solution volume on cellular response to CSE exposure. (**A**) NIH 3T3 mouse fibroblasts were plated at 10,000, 15,000, or 20,000 cells/well in a 48 well plate, allowed to grow for 24 hours, and damaged for 24 hours using 5% CSE at varying volumes (0.2 mL, 0.4 mL, 0.6 mL and 1.0 mL). An MTT cell viability assay was then used to assess relative cellular damage. **(B)** Cells were plated at 10,000 cells/well (**C**) 15,000 cells/well or (**D**) 20,000 cells/well, allowed to grow for 24 hours, and damaged for 24 hours using 0%, 4%, 5% or 6% CSE at varying volumes (0.2 mL, 0.4 mL, 0.6 mL and 1.0 mL). An MTT viability assay was then used to assess relative cellular damage. In each graph, values are reported as % viability compared to cells not exposed to CSE, with error bars representing the standard error of the mean.

Given the similar trends observed in the effect of cell count on the cellular response to CSE exposure in both 3T3 and NMuMG cells, we pursued further investigations with only the 3T3 cells. Previous reports suggesting a multitude of ways in which chemical bioavailability can be altered in the context of an in vitro assay [19, 20]. We hypothesized that cellular response to CSE would be dependent on the presence of critical soluble components of CSE that must be present at some minimal amount relative to the number of cells in order for cellular damage in the entire cell population to occur. Therefore, we theorized that increasing the amount of these soluble CSE components by simply increasing the total volume of CSE added to each sample would increase the apparent potency of the CSE by shifting the bioavailability of these components [19]. To test this hypothesis, we treated three different 3T3 cell counts (10,000, 15,000 or 20,000 cells) with different volumes of 4%, 5%, or 6% CSE, and confirmed that exposure of cells to larger volumes of CSE result in lower cell viability than exposure to smaller volumes of CSE (**Fig 3A–3D**). Interestingly, this volume dependent damage occurred despite the protection conferred by higher cell counts, suggesting that the two variables may counteract one another. Again, this is consistent with previous studies, suggesting that cellular response is dependent on the ratio of bioavailable chemical per cell [19].

### The bioavailability of soluble CSE components is limited by interactions between CSE components and both cells and other physical factors

**Fig 4:**
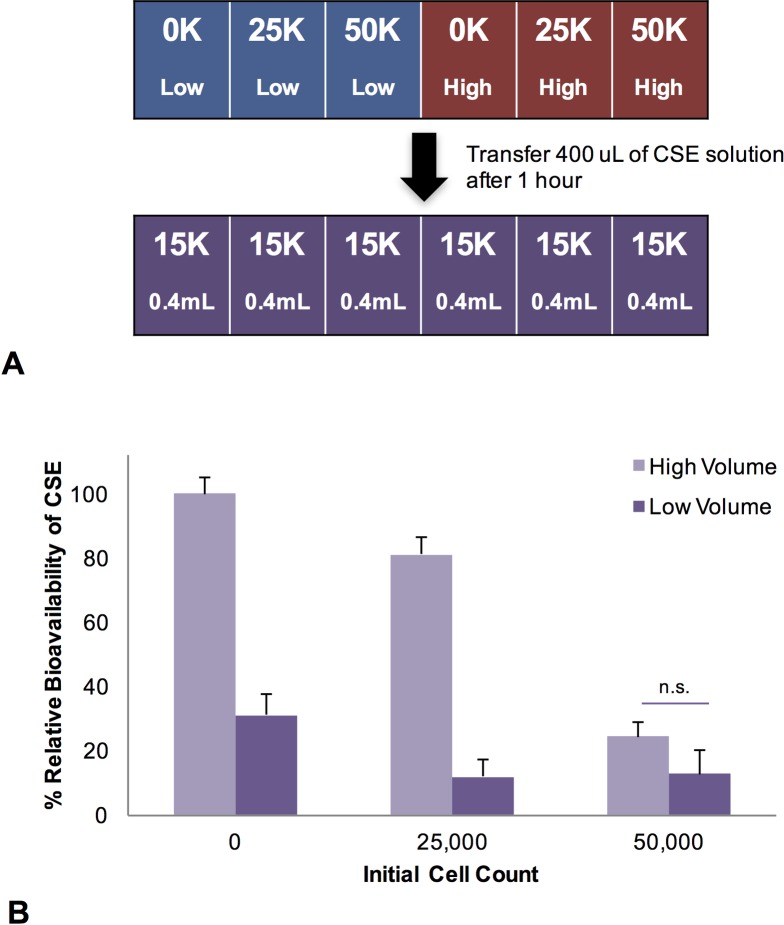
Relative bioavailability of CSE soluble components after exposure to different cell counts. (**A**) In this transfer assay, different cell numbers (0, 25,000; 50,000 cells/well) were exposed to one of two volumes of CSE (0.5 mL or 1 mL) for 60 minutes. After this short exposure, 400 uL of the resulting CSE solution was removed from the wells, and added to new undamaged cells (15,000 cells/well). These cells were exposed to the CSE solution for an additional 24 hours, before (**B**) analysis by MTT, and calculation of relative bioavailability compared to 100% bioavailability defined as equivalent to the cellular response to the amount of CSE available after transfer from wells with a high volume of CSE solution, and no cells present. Error bars represent the standard error of the mean. n.s. represents a lack of significance compared to other designated experimental conditions, p > 0.05.

The combined results discussed above suggest that there are soluble components in CSE that have limited bioavailability to cells in this in vitro assay. To further investigate the role of cellular interactions with CSE on the subsequent bioavailability of active soluble components, we performed a transfer assay outlined in the flow chart depicted in **Fig 4A**. In this transfer assay, different volumes of CSE (0.5 mL or 1 mL) were exposed to different cell numbers (0, 25,000; 50,000 cells/well) for 60 minutes. After this short exposure, 400 μL of the resulting CSE solution was removed from the wells, and added to new undamaged cells (15,000 cells/well). These cells were exposed to the transferred CSE solution for an additional 24 hours, before analysis of cell viability using the MTT assay. This assay is designed to assess the role of cellular interactions with CSE components, without specifically measuring the contributions from unknown physical factors (such as plastic binding, serum component binding, degradation or evaporation) [19,21]. Therefore, we set 100% CSE bioavailability equivalent to the cellular response after exposure to CSE transferred from wells with a high volume of CSE solution, and no cells present. Furthermore, we did not measure the impact of physical factors (plastic binding, serum component binding, degradation, evaporation) on CSE bioavailability at this high volume. However, we did find the role of physical factors is volume dependent. A significant loss in CSE bioavailability was observed in wells with a low volume of CSE solution, even when no cells were present, compared to the CSE bioavailability when used at high volume.

As mentioned, this transfer assay was designed to uncover the role of cell interactions with CSE components in limiting CSE bioavailability. Importantly, our data show that at high volumes, CSE bioavailability was severely limited by initial dose-dependent exposure to cells, as well as through a combination of cell-independent physical factors (**Fig 4B**). Our results also show that initially higher volumes of CSE retained a higher degree of bioavailability compared to lower volumes of CSE exposed to the same initial cell count. Therefore, this assay demonstrates that the initial interaction of CSE with cells is dependent on cell number, suggesting that cell number limits the bioavailability of the pertinent soluble components.

### Increased release of the cytotoxicity biomarker lactate dehydrogenase is dependent on both nominal CSE concentration and cell count

**Fig 5:**
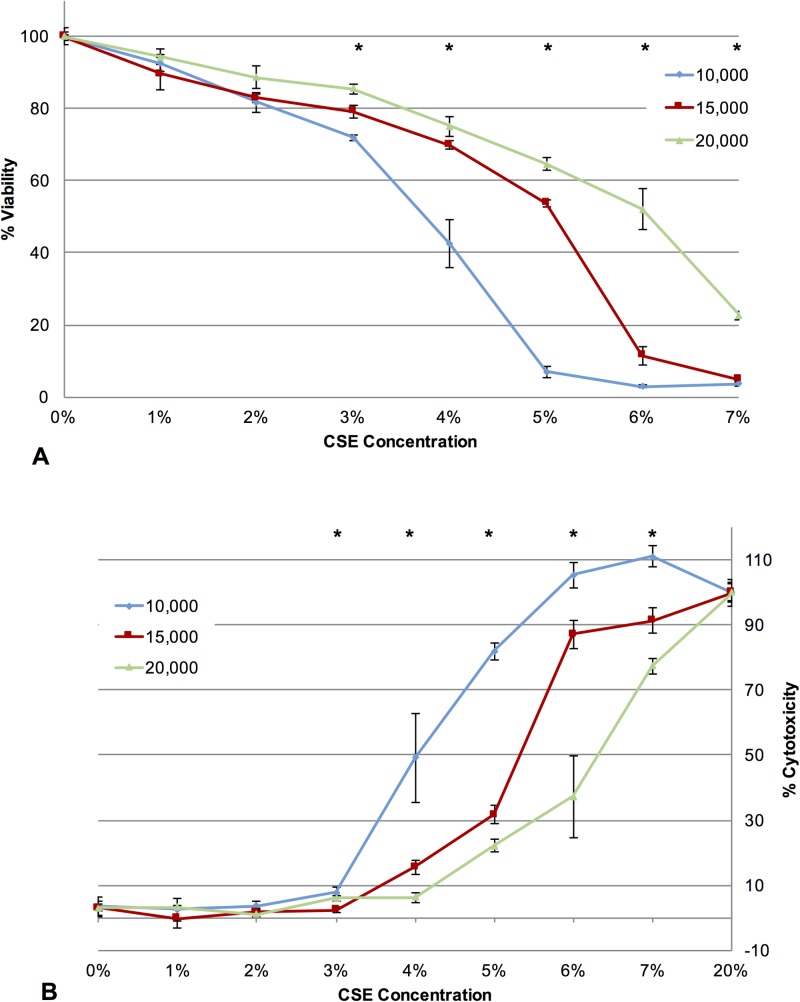
Effects of cell count on cytotoxicity biomarkers after CSE exposure, using the LDH release assay. (**A**) NIH 3T3 mouse fibroblasts were plated at 10,000, 15,000, or 20,000 cells/well in a 48 well plate, allowed to grow for 24 hours, and and exposed to CSE for 24 hours using 400 uL of 0–7% CSE. An MTT viability assay was then used to assess relative cellular damage. Values are reported as % viability compared to cells not exposed to CSE **(B)** Cells were plated and exposed to CSE as stated above, then a lactate dehydrogenase assay was performed to assess the amount of lactate dehydrogenase released. Values are reported as % cytotoxicity compared to cells exposed to 20% CSE (which was considered maximum LDH release), with error bars representing the standard error of the mean. * Compared to other cell counts exposed to the same percent CSE, p < 0.05.

In addition to cell viability, we predicted that the cellular response to CSE exposure could also be measured by examining biomarkers of cytotoxicity. In particular, release of lactate dehydrogenase from cells has been demonstrated to be a reliable marker of cellular cytotoxicity [22]. Therefore, we examined how cellular release of LDH is affected by changes in CSE nominal concentration and cell count. We examined the effect of cell count on the cellular release of LSH after CSE exposure by exposing either 10,000, 15,000, and 20,000 3T3 cells to varying concentrations of CSE (0–7%). After 24 hours of CSE exposure, we measured the amount of LDH release compared to maximal LDH release by those cells (**Fig 5A**). We also compared LDH release to the viability of the cells after damage to an undamaged control using the MTT assay (**Fig 5B**). the LDH release assay confirmed that higher cell numbers showed less evidence of cellular cytotoxicity compared to lower cell numbers at the same nominal CSE concentration.

### Variations in cell response to changes in CSE volume and cell count are also reflected in changes in mRNA transcripts of cellular cytotoxicity biomarkers

**Fig 6:**
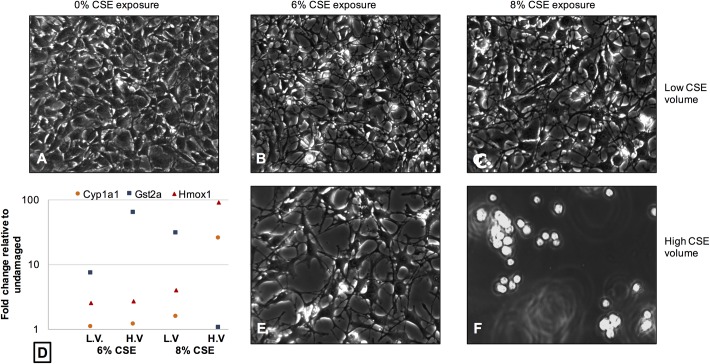
Morphological changes and altered expression of mRNA transcripts associated with cellular cytotoxicity are evident in the cellular response to CSE exposure for cells plated at 0.5 x 10^6^ cells/plate. Phase contrast images of (**A**) Undamaged cells (**B**) Cells exposed to 6% CSE at low volume (3 mL) (**C**) Cells exposed to 8% CSE at low volume (**D**) A scatterplot illustrating the general upward trend in expression of cellular cytotoxicity biomarkers at both CSE concentrations (6%, 8%) at either low volume (LV) or high volume (HV) (please see Table 2 for more details) (**E**) Cells exposed to 6% CSE at high volume (6 mL) (**F**) Cells exposed to 8% CSE at high volume.

**Fig 7:**
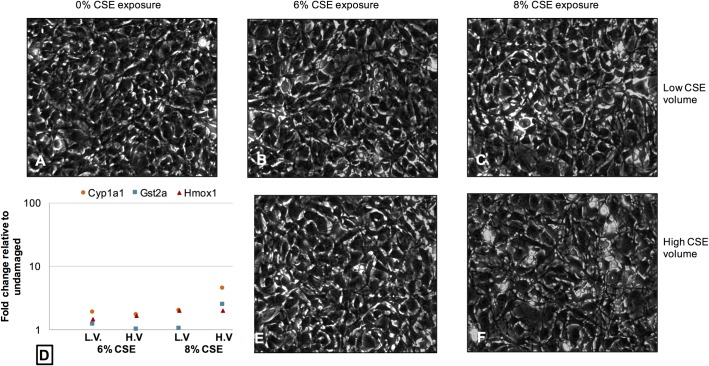
Morphological changes and altered expression of mRNA transcripts associated with cellular cytotoxicity are evident in the cellular response to CSE exposure for cells plated at 1 x 10^6^ cells/plate. Phase contrast images of (**A**) Undamaged cells (**B**) Cells exposed to 6% CSE at low volume (3 mL) (**C**) Cells exposed to 8% CSE at low volume (**D**) A scatterplot illustrating the general upward trend in expression of cellular cytotoxicity biomarkers at both CSE concentrations (6%, 8%) at either low volume (LV) or high volume (HV) (please see Table 2 for more details) (**E**) Cells exposed to 6% CSE at high volume (6 mL) (**F**) Cells exposed to 8% CSE at high volume.

To assess the cellular response at a molecular level, quantitative PCR was performed to analyze mRNA transcripts previously reported to be up-regulated in response to damage by cigarette smoke, including Cyp1a1, Gst2a and Hmox1 [23]. We also examined the relative expression of Ki67 as a marker of cell proliferation, as well as inflammatory markers that have been shown to be released from fibroblasts following exposure to CSE including Cox2/Ptgs2, Tfgβ and Ccl2/MCP-1 [16,24]. We examined the relative expression levels of these transcripts using 60 mm plates with two different cell counts (0.5 x 10^6^ cells/plate; 1 x 10^6^ cells/plate), two different concentrations of CSE (6%; 8%), and two different volumes (low volume– 3 mL; high volume– 6 mL). The results of this assay confirmed our observations from the cell viability assays and LDH release assay. Lower cell numbers, and cells exposed to higher volumes of CSE generally demonstrated higher expression, particularly of Cyp1a1, Gst2a, and Hmox1, and reduced expression of Ki67 (**Table 2**). Cellular changes were observed at all levels of CSE exposure in the low cell count (0.5 x 10^6^ cells/plate), demonstrated both by morphological changes in cell appearance, and a trend towards upregulation of cytotoxicity biomarkers at higher volumes of CSE, and higher concentrations of CSE (**Fig 6A–6E**). For example, at 0.5 x 10^6^ cells/plate, the cellular response to the highest CSE exposure (8% CSE, high volume) was demonstrated morphologically by uniformly rounded detached dying cells, with highly up-regulated expression of both Cyp1a1 and Hmox1. In contrast, at 0.5 x 10^6^ cells/plate the cellular response to the lower CSE volume of the same nominal concentration (8% CSE, low volume) was less severe, demonstrated morphologically by cells that were less rounded and still attached, with lower levels of both Cyp1a1 and Hmox1 compared to the high volume sample. At the higher cell count of 1 x 10^6^ cells/plate, no cellular changes were observed morphologically, even at the highest CSE exposure (8% CSE, high volume) (**Fig 7A–7E**). Interestingly, the general trend in expression of cytotoxicity biomarkers was still present, showing up-regulation at higher nominal concentrations of CSE as well as higher volumes, although the trend was at a lower level compared to the lower cell count. These data support our previous observations.

**Table 2:** Expression of select genes in cells exposed to different experimental conditions relative to cells not exposed to CSE.

		6% CSE		8% CSE	
		Low volume	High volume	Low volume	High volume
500K	Cyp1a1	1.1	1.2	1.54	*25.38
	Gst2a	*7.33	*63.95	*30.57	1.07
	Hmox1	*2.51	*2.70	*4.05	*90.13
	Ki67	*-2.05	*-2.87	*-3.25	*-5.12
	Ptgs2/Cox2	*1.58	*1.67	*2.33	*5.74
	Tgf0β	-1.18	1.17	*1.88	1.31
	Ccl2/Mcp1	-1.03	*-1.93	-1.07	*-2.60
1000K	Cyp1a1	1.86	1.73	*1.98	*4.55
	Gst2a	1.22	1.03	1.07	*2.48
	Hmox1	1.47	1.67	*2.01	*1.99
	Ki67	*-2.09	*-3.91	*-7.62	*-2.65
	Ptgs2/Cox2	1.34	1.13	1.23	-1.25
	Tgfβ	-1.42	-1.43	*1.53	-1.38
	Ccl2/Mcp1	1.26	-1.5	*1.70	-1.48

The numbers indicate the fold change in mRNA levels of each gene in cells exposed to various experimental conditions relative to the same cell count not exposed to CSE.

* indicates a significant difference compared to the same cell count not exposed to CSE (p < 0.05).

Cyp1a1 is a member of the cytochrome P450 family, needed to initiate metabolism of xenobiotics. In this assay, Cyp1a1 expression in cells exposed to CSE was not significantly up-regulated at the lower cell count (0.5 x 10^6^ cells/plate) until exposure to higher volumes of CSE at 8%, where it was dramatically up-regulated. Similarly, up-regulation of Cyp1a1 at the higher cell count (1,000,000 cells/plate) also occurred significantly at the high volume of 8% CSE, but not as dramatically as at the lower cell count. Gst2a is a member of the glutathione S transferase family needed for the subsequent phase of xenobiotic metabolism. Gst2a is dramatically up-regulated at the lower cell count (0.5 x 10^6^ cells/plate) after exposure to both concentrations of CSE (6% and 8%), increasing as much as 63 fold relative to baseline after cells were exposed to the high volume of 6% CSE. Interestingly, the expression of Gst2a is reduced to baseline when cells were exposed to the higher volume of 8% CSE, which may be associated with the overwhelming degree of cell death present at this exposure. Hmox1 is an enzyme induced by oxidative stress, which is thought to have anti-apoptotic functions (Grochot-Przeczek et al., 2012). In this assay, Hmox1 was significantly up-regulated at every CSE exposure condition, but was up-regulated more at higher concentrations and higher volumes of CSE for both cell counts. Hmox1 was dramatically up-regulated at the lower cell count (0.5 x 10^6^ cells/plate) at the highest volume of 8% CSE, under conditions in which the cells were morphologically rounded and largely unattached from the plate. Ki67 is a marker of cell proliferation, and was significantly and gradually down-regulated for both cell counts at each CSE exposure condition, but was generally down-regulated more at higher volumes of each CSE concentration. Of the inflammatory markers that we examined, only Ptgs2 and Tfgβ demonstrated trends with higher levels of expression at higher CSE volumes for a given cell count.

## Discussion

To better understand the cellular response to cigarette smoke in the pathophysiology of a wide variety of smoking-related diseases, many researchers utilize an in vitro assay in which cultured cells are exposed to a nominal concentration of soluble cigarette smoke extract (CSE). Such in vitro assays have been used to draw conclusions about cell death, cell phenotype changes, and cellular repair [16–18]. However, the experimental conditions used in these assays are variable, including not only the nominal concentration of CSE used, but also the cellular responses that are reported.

In this study, we note that only using the nominal concentration of CSE as a dosing metric is not an accurate method to predict cellular response after exposure to CSE. Specifically, we demonstrate that both CSE volume, as well as total cell number, affects the cellular response to CSE exposure. Exposure of cells to higher volumes of CSE (given at the same nominal concentration) results in more damage. Moreover, exposure of greater numbers of cells to the same nominal concentration of CSE results in less damage. These phenomena can be replicated using a variety of assays including measures of cell viability, and of cellular cytotoxicity biomarkers. In this study, we generated CSE by drawing cigarette smoke through DMEM media as previously reported [24,25], and stored the CSE solution at -80C until use [25–27]. We verified that the effects of CSE on cell viability were not significantly altered by freezing using an MTT assay. Future comparison of specific pro-inflammatory, toxicological, pro-proliferative, and extracellular matrix remodeling markers after cellular exposure to fresh versus frozen CSE will further add to the understanding of how frozen CSE can be used in a variety of toxicological, pathological and inflammatory assays.

Work with other in vitro xenobiotic assays suggests that, although not yet documented for CSE, the observations we report here are not uncommon [19,21]. A recent study by Doskey *et al* noted that both volume and cell number effects cell viability after exposure to two model compounds 1,4-benzoquinone or oligomycin A [19]. These authors and others have suggested that bioavailability, as defined by the fraction of test chemical that is actually available for uptake into cells, cannot be accurately described using the nominal concentration of the chemical [19,20]. Suggested reasons for this discrepancy include reductions in the freely available test compound through degradation, metabolism by cells, cell binding, media interactions, or binding to plastics [20,21]. In this study, all cells were maintained in DMEM media containing 10% FBS, which is standard for optimal growth of 3T3 and NMuMG cell lines. To maintain consistent growth in our assays, DMEM with 10% FBS was also used for both the control and CSE exposure groups. The presence of FBS may affect both cell proliferation and CSE bioavailability, and should be a consideration in assay development.

**Fig 8:**
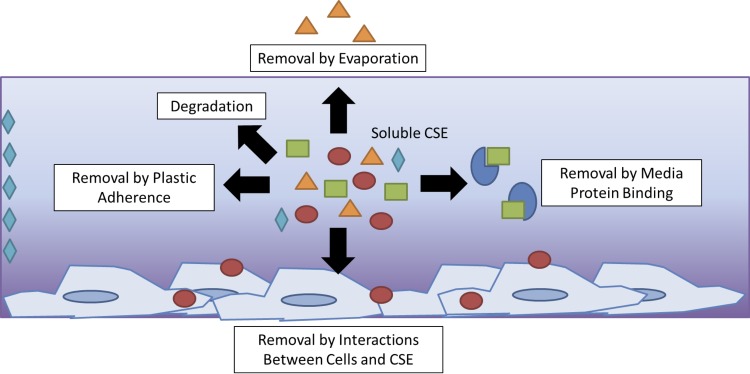
Modeling CSE bioavailability during cellular in vitro exposure. As illustrated, there are several different ways in which CSE bioavailability may be limited, including binding of CSE components to cell culture plastics, serum factors, or interactions with cells. Furthermore, CSE bioavailability may be reduced through either degradation or evaporation of CSE components.

In many cases, the effect of cellular interactions with the test compound can be significant enough to reduce the bioavailability of the compound to other cells [21]. Our study suggests that this is equally applicable to adherent cell-based in vitro assays using CSE. In particular, we performed a unique transfer assay in which different volumes of CSE were exposed to different cell counts for 60 minutes. After this short exposure, a standard volume of the resulting modified CSE was removed from each well, and added to new cells. These cells were exposed to the resulting CSE solution for an additional 24 hours, before analysis of cell viability using the MTT assay. Our results show that CSE initially exposed to higher cell numbers had lower bioavailability after transfer, compared with CSE exposed to lower cell numbers. In addition, initially higher volumes of CSE retain a higher bioavailability compared to lower volumes exposed to the same initial cell count. These data suggest that cell number limits the bioavailability of the pertinent soluble components, potentially through cell-binding or cellular metabolism mechanisms mentioned in previous studies, and as illustrated in **Fig 8** [20,21]. Although the effects of other physical factors on CSE bioavailability was not specifically measured in this assay, we did observe that CSE bioavailability after transfer was retained to a greater degree when a high volume (1 mL) of CSE was initially exposed to wells (with no cells) compared to exposure of a lower volume (0.5 mL) of CSE. This suggests that physical factors do indeed play a role in CSE bioavailability, and may include binding of CSE components to the plastics in the cell culture wells, evaporation and degradation, as illustrated in **Fig 8**.

Measures of both cell viability using the MTT assay, and of cellular cytotoxicity using the LDH release assay confirm that experimental variables such as cell count and CSE volume can alter the cellular response after exposure to CSE. Under the experimental conditions described in this study, the measurement of LDH release was only feasible to investigate the effect of cell count on cellular response to CSE. The effect of CSE volume on cellular response cannot be measured using this assay, since the LDH released will be diluted into different volumes of liquid. Moreover, the CSE bioavailability assay is also not appropriate for measuring LDH release, since the cells that are initially exposed to CSE will release LDH that will then be transferred to the second set of cells. However, in addition to LDH release analysis of mRNA transcripts for cytotoxicity biomarkers also confirmed the trends demonstrated in this study.

We assessed the relative expression of several genes previously reported to be up-regulated in fibroblasts in response to CSE-induced damage [23]. In particular, we examined the relative expression levels of genes involved in xenobiotic metabolism (Cyp1a1, Gst2a, Hmox1) that are markers cellular cytotoxicity, as well as cell proliferation (Ki67), and inflammatory response (Ccl2/MCP1; Cox2/Ptgs2; Tgfβ). The results of this assay confirmed what we observed in both the cell viability and LDH release assay. Lower cell numbers were damaged more (higher expression of Cyp1a1, Gst2a and Hmox1; lower expression of Ki67), and cells exposed to higher volumes of CSE were also damaged more. In particular, the expression of Cyp1a1, a member of the cytochrome P450 family needed to initiate metabolism of xenobiotics, was up-regulated as cell damage increased. Importantly, this occurred most dramatically at the lowest cell count, highest volume, and highest nominal concentration, effectively demonstrating the interplay between these variables.

Gst2a, a member of the glutathione S transferase family needed for the subsequent phase of xenobiotic metabolism, is also drastically up-regulated in cells damaged by CSE. In both cases, the up-regulation of Gst2a was seen for most conditions of CSE used for exposure to the lower cell count, but not seen in most CSE conditions used for exposure to the higher cell count. These data corroborate our previous observations that higher cell numbers experience less damage under the same CSE conditions compared to lower cell numbers. Interestingly, at levels of very severe damage resulting in almost total cell death (seen for the lower cell counts exposed to high volumes of 8% CSE), the expression of Gst2a actually drastically declined. We speculate that this dramatic drop is consistent with the known diverse roles of glutathione S transferases in the cell. GSTs are generally anti-apoptotic, via complex formation with JNK. However, under conditions of extreme oxidative stress, these complexes are dissociated, and GST is oligomerized, allowing apoptosis of the cell to occur [28]. Down-regulation of GST gene expression has also been similarly reported in the context of conditions that increase apoptosis, as seen in our assay [29].

Hmox1 is gradually up-regulated across most exposure conditions at the lower cell count, and very dramatically up-regulated at exposure to the highest volume of the highest nominal concentration of CSE. Conversely, at the higher cell count, the relative expression levels of Hmox1 are much lower. Hmox1 is a member of the heme oxygenase family of proteins, and is responsible for converting heme into bilirubin. Heme oxygenases are transcriptionally up-regulated by agents that increase oxidative stress, and the metabolite byproduct of heme metabolism, bilirubin has been described as a powerful anti-oxidant [30]. We speculate that the dramatic up-regulation of Hmox1 during cell death is consistent with its previously described role. Ki67, a marker for cell proliferation, steadily declines across both cell counts, and all CSE exposure conditions, again consistent with our previous observations. We also measured expression of three inflammatory markers that have been previously reported to be associated with the response of fibroblasts to CSE exposure, including Cox2/Ptgs2, Tgfβ and Ccl2/Mcp-1 [16,24]. In this assay, expression of Ptgs2 and Tgfβ mRNA demonstrated trends towards upregulated mRNA expression at higher CSE bioavailability. However, none of these inflammatory markers were as sensitive to changes in CSE bioavailability as the cytotoxicity markers. It is possible that the release of inflammatory markers is complicated by the level of cellular cytotoxicity, and for some markers higher cytotoxicity maybe associated with reduced release of a particular inflammatory marker. In addition, expression of these inflammatory markers may be influenced by the local microenvironment. In the higher cell count plates, different interactions may occur between neighboring cells that might influence release of inflammatory markers.

The results of our study suggest that the cellular response to cigarette smoke is dependent on the ratio of bioavailable soluble toxins to cell number. If there is higher ratio of bioavailable toxin per cell through a combination of higher nominal concentration, higher volume of CSE, or lower cell number, the cells are damaged more. This has broad implications for the nature of cellular response to CSE exposure, and for the design of in vitro assays using CSE. In particular, our data suggests that interactions between CSE components and cells are potentially a major limiting factor in CSE bioavailability. These interactions could include several phenomena occurring between the xenobiotic and the cells, ranging from binding to the outside of the cells, to active metabolism of CSE components by the cells. We speculate that the non-linear reduction in CSE bioavailability may suggest a more complex interaction between the CSE and cells, including possible cooperative cell mechanisms to avoid damage from toxins. Recent work in computational biology suggests that cells acting as individuals may respond differently to external stimuli compared to cellular aggregates that can demonstrate cooperative crosstalk that result in changes in cellular metabolism and proliferation [31]. It is possible that at high cell numbers, CSE bioavailability is limited in a non-linear fashion by the presence of such aggregate cellular crosstalk mechanisms.

In conclusion our results highlight important variables that have previously been underappreciated in literature utilizing soluble CSE as an in vitro model for smoking diseases. We report that exposure of cells to CSE results in complex interactions that have yet to be described in this model. This identifies several interesting questions concerning how cells interact with cigarette smoke components both in vitro and in vivo. Furthermore, we propose that future studies using this model of CSE exposure make attempts to acknowledge the role of these variables by reporting not only the nominal concentration of CSE used, but also the number of cells used, the total volume of CSE/media used, and the specific cellular responses observed.
